# Starting life in space

**DOI:** 10.1093/nsr/nwaa102

**Published:** 2020-06-17

**Authors:** Jake Cornwall-Scoones, Magdalena Zernicka-Goetz

**Affiliations:** Division of Biology and Biological Engineering, Californian Institute of Technology, USA; Division of Biology and Biological Engineering, Californian Institute of Technology, USA

As far as we know, animal development is a process that is unique to our planet. That does not mean, however, that development beyond our realm is impossible. As it starts to become feasible for us to look to the sky for another place to call home, we may start to appreciate the gravity of this question.

In this issue, Lei *et al*. investigate for the first time the repercussions of space travel on the first decisions made by mouse embryos. Developing a novel micro-incubator, capable of automatic micrography and fixation, harboring some 3400 two-cell embryos, they investigate the consequences of development after being projected into the stratosphere in the SJ-10 satellite [1].

This is not the first foray in embryonic space travel. In the 1990s, *in vitro* fertilization of *Xenopus laevis* embryos on a space shuttle was used to demonstrate how cortical rotation, key in establishing bilateral symmetry, was independent of gravity [2]. But as the mammalian organism for which early development is best understood, studying the mouse provides perhaps the closest insight into the consequences of space on embryonic development of our own species.

The first decision made by cells of the mouse embryo is whether to become trophectoderm (TE) or inner cell mass (ICM), with only the latter having the potential to give rise to the cells of the adult, a decision made gradually, with biases emerging from as early as the four-cell stage [3–5]. The authors find that space travel alters the balance of ICM/TE commitment, biased towards ICM and transitional, uncommitted fates.

There are two fundamental differences in environment for embryos developing in space, namely radiation and microgravity. Simulated microgravity is found to have no significant impact on development, whereas gamma-radiation, even in very small doses, has substantive effects on embryo viability. In space, and when simulated on Earth, radiation is reported to promote widespread DNA damage, incurring both single- and double-stranded breaks. Investigating consequences through bisulfite sequencing, which surveys genome-wide DNA methylation [6], radiation is shown to promote shifts in cell state, associated with stress response and epigenetic modifications.

Beyond viewing this investigation as a feasibility study for life beyond Earth, these findings provide insight into how our developing embryos might respond to challenges such as this. It is known that early mouse development is remarkably plastic, capable of generating healthy adults after the aggregation of multiple embryos or upon removal of cells [7,8]. In spite of this plasticity, there are some crucial constraints. Notably, development is only viable if the embryo has a minimum of four epiblast cells (giving rise to adult tissues) upon implantation into the uterus [9]. A similar constraint in cell number is observed in assembling cellular rosettes that have to generate the pro-amniotic cavity, laying the ground-plan for gastrulation [10].

Could this bias towards ICM fate observed in this study be yet another example of plasticity in the face of constraint, generating embryos more likely to develop to term? Embryonic and extra-embryonic lineages respond differentially to genomic aberrations, with aneuploid TE cells slowing their proliferation and epiblast cells undergoing programmed cell death [11]. If radiation promotes a bias towards ICM, this could allow for greater survivorship in the context of cell damage and death. Probing the mechanistic consequences of epigenomic reprogramming in response to stress will be integral to addressing these questions, helping unveil deviations in fate specification both in space, but also importantly on Earth, where radiation may have equal impacts.

A second striking finding of this study is that while lineage allocation is altered, the morphology of the blastocyst remains unaffected. This observation highlights challenges of the gene-centric view of early embryonic self-assembly, contributing to evidence that blastocyst morphogenesis and cell-fate specification can be decoupled. While the effects of space are negligible in early morphogenesis, impacts on later morphogenesis at peri- and post-implantation stages remain unknown, where, conceivably, gravity may play a greater role.

By investigating pre-implantation mouse development in space, Lei *et al.* bring the prospect of life beyond Earth one step closer [1]. With the desire for long-term space travel only heightened by concerns of climate collapse, understanding how gravity and radiation could jeopardize initiation of a new life in the cosmos is crucial. Beyond life as we know it, the research helps throw light on potential adaptations of genesis in extra-terrestrial life. Through subjecting embryos to unworldly stresses, Lei *et al.* bring new perspectives on both life beyond our world, as well as life on it.
